# Genetic Testing Yield for Dilated Cardiomyopathy in a Single Lithuanian Center

**DOI:** 10.3390/diagnostics16132115

**Published:** 2026-07-06

**Authors:** Marius Šukys, Eglė Ereminienė, Kristina Aleknavičienė, Rimvydas Jonikas, Karolina Mėlinytė-Ankudavičė, Paulius Bučius, Rasa Ugenskienė

**Affiliations:** 1 Department of Genetics and Molecular Medicine, Medical Academy, Lithuanian University of Health Sciences, 50161 Kaunas, Lithuania; 2 Institute of Cardiology, Lithuanian University of Health Sciences, 50103 Kaunas, Lithuania; 3 Heart Centre, Medical Academy, Lithuanian University of Health Sciences, 50140 Kaunas, Lithuania

**Keywords:** dilated cardiomyopathy, genetic testing, *TTN*

## Abstract

**Background/Objectives**: Dilated cardiomyopathy is a heterogeneous disorder with a substantial genetic contribution from a variety of pathogenic variants. Hereditary isolated DCM is often caused by variants in genes encoding sarcomere proteins, as well as proteins involved in desmosomes or other cardiac cell functions. Identifying genetic causes improves our understanding of DCM pathophysiology, facilitates prognostic assessment, and enables more personalized disease management. **Methods**: We retrospectively analyzed genetic data from adult patients with a clinical diagnosis of isolated DCM evaluated at a Lithuanian tertiary university hospital between 2019 and 2024. All patients were tested with a next-generation sequencing cardiovascular gene panel. **Results**: We gathered 169 patients and initially reached a 16.0% (*n* = 27) genetic testing diagnostic yield. We performed all genetic variant reanalyses with the most current classification guidelines, and we found an additional eight positive cases. Our final diagnostic yield was 20.7% (*n* = 35). *TTN* was the most frequently affected gene (*n* = 30), whereas variants in *BAG3* (*n* = 2), *DSP* (*n* = 1), *LMNA* (*n* = 1), and *FLNC* (*n* = 1) were rare. In total, 15 variants were novel—not described in the literature or databases. We did not observe significant clinical differences between patients with pathogenic variants and those without pathogenic variants. We expected a different clinical course with variants in genes like *BAG3* or *LMNA*, but there were only a few cases. **Conclusions**: Genetic testing remains an important tool for confirming complex DCM cases and allows earlier disease management for relatives at risk.

## 1. Introduction

Dilated cardiomyopathy is a major public health burden as it is one of the leading indications for heart transplantation and a major cause of heart failure [1]. The defining feature of DCM is left ventricular dilatation with reduced function in the absence of hypertension, valvular, congenital, or ischemic heart disease [2,3]. DCM has both genetic and non-genetic causes, and this heterogeneity makes it difficult to determine its true prevalence. Non-genetic DCM may result from drug use, exposure to toxins, abuse of alcohol, viral and bacterial infections, or autoimmune disorders, may be related to other conditions such as pregnancy, or may be the combined effect of all mentioned factors [1]. In addition to monogenic causes, syndromic forms such as muscular dystrophies, mitochondrial disorders, and metabolic diseases should also be considered.

Reported prevalence ranges from approximately 1 in 250 to 1 in 2700 individuals [4,5], which possibly reflects various causes of DCM. A UK Biobank analysis searching only isolated monogenic DCM found a prevalence of about 1 in 220 [6]. Interestingly, pathogenic *TTN* variants have been identified in approximately 1 in 250 adults without known heart failure [7]. Not all DCM cases may be recognized clinically, partly due to age-dependent penetrance.

Establishing a diagnosis of DCM often requires comprehensive evaluation to identify the underlying cause and enable targeted treatment [8]. This includes many laboratory tests like checking calcium, ferritin, complete blood count, liver, thyroid, renal function, and troponin. Depending on suspicion and age of onset, additional tests can include secondary metabolic screening such as carnitine profile, free fatty acids, and antibody testing. Confirmation of ventricular dilatation and systolic dysfunction requires imaging studies such as echocardiography and cardiac magnetic resonance imaging. These evaluations should also be offered to first-degree family members to identify familial cases. In selected cases, suspicion of myocardial infiltration or inflammation may require endomyocardial biopsy. Finally, genetic evaluation may help confirm a hereditary etiology.

Isolated hereditary DCM is most commonly caused by variants in sarcomere-encoding genes, particularly *TTN*. Rare variants in at least 12 genes have been shown to be enriched in DCM and are considered potentially causative [9]. Haploinsufficiency of sarcomere genes leads to the reduced expression of proteins such as titin, myosin, actin, and troponin [10]. Also, protein deficiency can increase metabolic demand, which further facilitates eccentric heart remodeling [11]. The involvement of other genes is more complex; for example, lamin deficiency causes nuclear envelope distortion, especially in contractile cardiomyocytes. Lamin is coded by *LMNA*, and cardiomyopathy associated with this gene presents with a more severe phenotype, with higher incidence of malignant arrhythmias and a need for heart transplantation [12]. Variants in *DSP*, which encodes desmoplakin, can also cause a distinct phenotype with high arrhythmic burden, variable left ventricle ejection fraction, and left ventricle scarring [13]. Loss of desmoplakin disrupts intercellular adhesion, which allows easier fibrosis formation. *BAG3* is one of the more recently established genes associated with DCM [14]. Its protein has complex functions, including the stabilization of myofibril structure under stress [15]. Its phenotype presents great variability but fewer arrhythmia events are noted. These findings illustrate the diversity of genetic mechanisms and their associated phenotypic variability.

The first genetic cause of DCM was identified in 1998 by MT Olson et al. [16]. At that time, missense variants in the *ACTC1* gene were identified as the causative factor. Later studies based on this discovery tried to find more cases but were largely unsuccessful [17,18]. Now this gene is more commonly associated with hypertrophic cardiomyopathies and very rarely is it the cause of DCM [19]. The identification of causative variants may help confirm diagnosis in ambiguous cases or adjust patient care as some genetic variants lead to more pronounced conduction damage. In this study, we report our experience with genetic testing in patients with DCM at a tertiary university hospital.

## 2. Materials and Methods

### 2.1. Patient Selection

A retrospective study was performed between 2019 and 2024 at the Department of Genetics and Molecular Medicine, Hospital of the Lithuanian University of Health Sciences Kauno Klinikos. Ethical approval was granted by the Kaunas Regional Biomedical Research Ethics Committee (No. BE-2-63). Demographic, clinical, and genetic data were collected from electronic health records. Patients referred for genetic evaluation because of a confirmed or suspected diagnosis of dilated cardiomyopathy were included in the study. Clinical diagnosis was confirmed through echocardiography or cardiac MRI. We used the European Society of Cardiology (ESC) definition of dilated cardiomyopathy, which is characterized by a left ventricular (LV) end-diastolic diameter greater than 58 mm in males and greater than 52 mm in females, along with an LV end-diastolic volume index of 75 mL/m^2^ in males and 62 mL/m^2^ in females as measured by echocardiography. Left ventricular global systolic dysfunction is identified when the left ventricular ejection fraction (LVEF) is less than 50% [2]. All patients received genetic testing. We included only isolated non-syndromic cases (for example, patients with a primary skeletal muscle disorder which causes secondary DCM are excluded). We also excluded asymptomatic individuals who underwent genetic evaluation solely because of an affected family member had been identified.

Inclusion criteria:

- Age ≥ 18 years at the time of genetic consultation;
- Availability of cardiac imaging, including echocardiography and/or cardiac magnetic resonance imaging (MRI);
- Diagnosis or clinical suspicion of dilated cardiomyopathy established by a multidisciplinary team consisting of a clinical geneticist and cardiologists;
- Underwent genetic testing.

### 2.2. Cardiac Investigation

The cohort consisted of patients with a long-standing diagnosis of DCM as well as those who were newly diagnosed. Data collected included symptoms, age at onset, laboratory and instrumental findings, complications such as atrial or ventricular fibrillation, and treatments including cardioverter or pacemaker implantation, as well as mortality data. As not all patients had MRI scans performed, the ejection fraction was evaluated using the first available data from health records, regardless of whether it was measured by MRI or ultrasound. Other cardiac parameters were compared based on the imaging method used (MRI or ultrasound). MRI scans were conducted on a 3T magnetic resonance imaging scanner with an 18-channel cardiac coil (MAGNETOM Skyra, Siemens Healthcare, Erlangen, Germany). Images were obtained during expiratory breath-holding with ECG gating. In cases where MRI was not performed or no data were available, parameters from heart ultrasound were utilized. Two-dimensional (2D) transthoracic echocardiography (TTE) at rest was performed by an experienced echocardiographer using a diagnostic ultrasound system (EPIQ 7, Philips Ultrasound, Inc., Bothell, WA, USA). All measurements were taken in accordance with established guidelines. A family history was considered positive if first-degree relatives experienced sudden cardiac death before the age of 60 or if there were known cases of DCM within the family.

### 2.3. Genetic Investigation

Patients’ peripheral blood samples were collected with EDTA tubes. Genomic DNA extraction was performed via automatic workflow with QIA symphony (QIAGEN, Hilden, Germany) with the reagent kit DNA midi Kit, or with QIcube (QIAGEN, Hilden, Germany) with the reagent QIAmp DNA blood Mini Kit. Purity and concentration were assessed using Qubit (Thermo Fisher Scientific, Waltham, MA, USA) and dsDNA HS Assay kit reagents. Next-generation sequencing was performed with the Sistemas Genómicos (Valencia, Spain) Cardio-GeneSGKit library preparation kit and the Illumina NextSeq 550 system (San Diego, CA, USA). The Cardio-GeneSGKit library gene list was updated during the study period, but genes important/established for the DCM were not compromised. Detailed lists are in the Supplementary Materials (S1 list—older panel; S2 list—newest panel). All laboratory procedures were conducted in accordance with the manufacturers’ instructions. Sequencing achieved an average depth of 200 times per nucleotide. The genetic test was repeated when it did not achieve the targeted depth. Most target genes achieved complete coverage. Variant analysis and reporting were restricted to coding exons and flanking intronic regions extending 20 bp from exon–intron boundaries.

Sequencing reads were aligned against human reference genome version GRCh38. Read alignment was performed using the Burrows–Wheeler algorithm (BWA). After read mapping, low-quality reads and sequences flagged as PCR duplicates were removed. Variant calling was performed using the Genome Analysis Toolkit (GATK). Low-quality variants (coverage < 10×, allele balance < 20%, Phred < 20) were removed. We selected only those genes for analysis that showed evidence of causing DCM by PanelApp: *TNNT2*, *ANKRD1*, *DSP*, *NEXN*, *DSG2*, *ACTC1*, *MYBPC3*, *RBM20*, *DES*, *SCN5A*, *LDB3*, *VCL*, *FLNC*, *JUP*, *TCAP*, *FKRP*, *MYH6*, *TNNI3*, *PKP2*, *EMD*, *TBX5*, *BAG3*, *SGCD*, *TMEM43*, *CSRP3*, *FKTN*, *ACTN2*, *TNNI3K*, *TTN*, *MYPN*, *GATA6*, *TPM1*, *LAMP2*, *CRYAB*, *PLN*, *TNNC1*, *DMD*, *MYH7*, *TBX20*, *DSC2*, *NKX2-5*, *ANK2*, *RYR2*, *PRDM16*, and *LMNA* [20]. Variants were initially classified according to the 2015 ACMG/AMP [21] and relevant ClinGen recommendations. Given the retrospective nature of the study and the evolution of variant interpretation criteria over time, all identified variants were re-evaluated using the most recent gene-specific ClinGen recommendations [22]. Variants of uncertain significance (VUS) were excluded from the present analysis; however, clinically actionable variants were communicated to patients as part of routine clinical care.

### 2.4. Statistical Analysis

Cases were coded and research data were stored in a Microsoft Office Excel spreadsheet. Statistical analysis was performed using nonparametric tests like Chi square, the Mann–Whitney U test, and Spearman, as data was not normally distributed (confirmed with the Kolmogorov–Smirnov test). Statistical calculations were performed using IBM SPSS 26.0 software.

## 3. Results

Data from 169 patients with dilated cardiomyopathy phenotype were collected, of whom 69.8% were males and 30.2% females. The mean age at genetic consultation was 51.54 ± 12.26 years, with males tested at a slightly older age than females (51.80 ± 12.02 vs. 50.97 ± 12.90; *p* < 0.05). Pathogenic (P) or likely pathogenic (LP) variants in one of the DCM-causing genes were identified in 20.7% of cases (*n* = 35) (Table 1). The detection rate was similar between males and females (21.2% vs. 19.6%; *p* = 0.054). Patients carrying pathogenic variants were slightly younger at the time of testing (49.63 ± 11.93 vs. 52.05 ± 12.34) and more frequently had affected first-degree family members (27.8% vs. 19.2%; *p* = 0.266).

**Figure 1:**
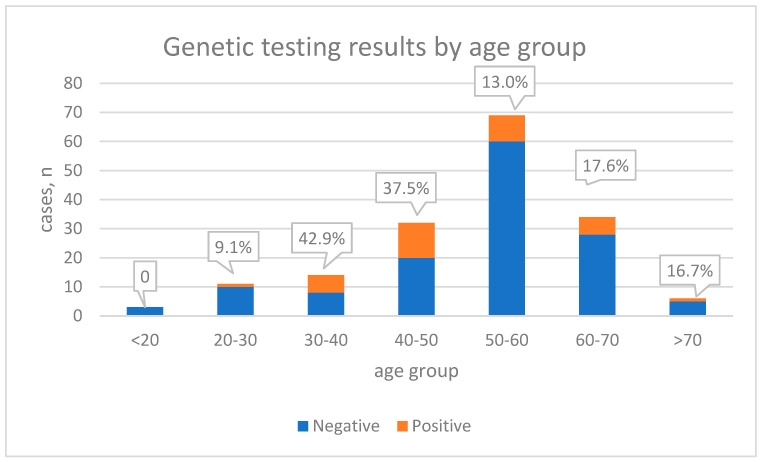
Cohort presented by age group and percentage of positive genetic testing.

Patients were divided into two equal groups based on age: younger and older than 54 years. Comparing clinical parameters or genetic testing results, we found no differences. When stratified by decade, the highest proportion of pathogenic or likely pathogenic (P/LP) variants was observed in patients aged 30–40 and 40–50 years (42.9% and 37.5%, respectively) (Figure 1). Patients younger than 54 years with a positive family history had a significantly higher probability of carrying a causative variant than all other patients (40% vs. 18.1%; *p* = 0.023; Chi square). These patients also had higher LVEF (36.86 ± 12.47 vs. 30.20 ± 11.78; *p* = 0.027; Mann–Whitney).

Patients with and without pathogenic variants had similar ejection fraction (32.09 ± 12.99 vs. 30.70 ± 11.80; *p* = 0.595). After at least one year of follow-up, ejection fraction improved similarly in both groups (*n* = 130; 36.12 ± 11.67 vs. 34.94 ± 12.21; *p* = 0.57). When comparing other clinical parameters, such as left ventricle end-diastolic volume/diameter, arrhythmias, and complications, we found no differences. Clinical features are summarized in Table 2.

**Figure 2:**
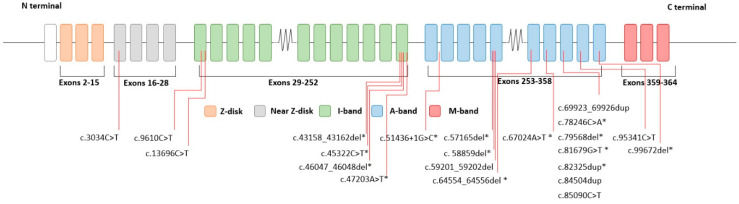
Schematic representation of *TTN* gene and P/LP variants found. Many variants are found in distal half of *TTN* gene. Variants marked with * are novel. Regions marked using Cardiodb data [23].

Most patients carried *TTN* gene pathogenic variants—30 (16 nonsense, 13 indels with frameshift, and 1 splice site). Two patients had pathogenic variants in *BAG3*, and single cases were identified in *DSP*, *FLNC*, and *LMNA*. All variants were reanalyzed according to current guidelines, resulting in 8 additional patients receiving a positive genetic diagnosis (included in the total 35 cases). Six of these variants are found in the I-band of titin, but their exon percentage spliced-in is 100%. Overall, 10 *TTN*-positive cases affected the I-band and 20 the A-band; however, this did not correlate with clinical phenotype. In total, 15 variants were novel - not described in the literature or clinical databases. *TTN* variants c.13696C>T and c.69923_69926dup were found 5 and 4 times. The recurrence of these variants suggests that they are unlikely to be de novo; however, no affected family members were reported. *De novo* status could not be assessed, as many patients’ parents were deceased. The *TTN* gene 326 exon was found to be the most commonly affected (*n* = 11). No statistically significant differences were observed between patients with variants in exon 326 and those with variants in other regions of *TTN*. *TTN* affected regions are marked in Figure 2. All detected variants are presented in Table 3.

Among *TTN*-positive patients, those with frameshift variants showed a higher prevalence of ventricular arrhythmias (4 vs. 0 cases) and a greater need for implantable devices (6 vs. 1 cases) compared to those with nonsense variants; however, these differences did not reach statistical significance.

## 4. Discussion

We present our experience with genetic testing in patients with DCM at a tertiary university hospital. Our patient selection criteria were relatively broad, as individuals with suspected DCM were also included (following multidisciplinary team decision). Despite that, we achieved a 20% genetic testing diagnostic yield, which is consistent with reports from other centers [9,24,25,26]. Mazzarotto et al. showed that patients selected by specialists have a higher diagnostic yield: 17% in unselected DCM cases vs. 26% with diagnostic referral (with early-onset disease or enriched familial history) [9]. It remains unclear whether offering genetic testing to a broader population is beneficial, as many individuals carrying pathogenic sarcomere gene variants do not develop cardiomyopathy. Thus, unselected patients with DCM might not benefit from genetic testing as their disease might not be necessarily managed differently. It has been proposed that DCM can be divided into three entities (risk of DCM; asymptomatic clinical DCM; symptomatic DCM), which would explain why some patients with pathogenic variants do not develop DCM [27]. Part of this cohort was analyzed previously for cases without P/LP in order to search for additional polymorphisms which could explain phenotype variability [28]. Disease severity was associated with variant burden in genes *GATAD1*, *LOX*, *RASA1*, *KRAS*, and *KRIT1*, but this finding could be accidental.

Although *MYH7* is described as the second most common cause of hereditary DCM, we did not identify any pathogenic variants in this gene in our cohort [29]. Previously, we described our cohort with hypertrophic cardiomyopathies, and *MYH7* was the second most common causative gene [30]. DCM caused by *MYH7* is described with less frequent AF and atrioventricular conduction problems than other DCMs, which may lead to reduced healthcare-seeking behavior. Such behavior may contribute to the lower detection rate of *MYH7*-associated DCM in our cohort. However, this inference remains speculative.

Patients with *BAG3* P/LP variants are reported to have a lower arrhythmogenic risk and possibly more rapidly deteriorating left ventricle function [31]. Our case involving *BAG3* c.514c>t has been previously published with unusually early manifestation at 22 years old with AF [32]. The same was found for his father—early onset with AF. Our other case was with variant c.1385T>C, which we classified as a variant of unknown significance. This variant is reported multiple times with DCM cases [33], and despite not reaching P/LP criteria, we reported it as causative for the patient. The patient presented with symptoms at 44 years old and had ventricular tachycardia episodes. These two cases confirm early onset, but arrhythmias may also occur.

A hallmark of hereditary DCM is truncating variants in the *TTN* gene. Despite *TTN* coding the biggest protein in humans and having distinct regions with different functions, clinical variability is generally not strongly associated with the location or type of *TTN* gene variant [34]. Similarly, we did not observe significant phenotypic differences among *TTN*-positive patients.

Previous analyses of clinical significance of *TTN* genetic variants were focused on A-band titin protein [22]. This was possibly an oversimplification of the titin pathogenicity mechanism, as variants in other locations have been described as causative [34]. The *TTN* I-band region is extensively spliced, but not every exon is affected equally, as it has been shown that some I band exons have a high percentage spliced-in (PSI) score in cardiac muscle cells [23,35]. We reanalyzed our genetic variants according to PSI, which resulted in the reclassification of *TTN* variants to P/LP for eight patients, seven of them in the I-band. The variant c.13696C>T reoccurred in five of our unrelated cases and is described in the literature from 2012 [36]. Gnomad V4.1.1 shows that the variant appears only in the European non-Finnish population (*n* = 10), which may suggest a potential founder effect in this population [37].

We found that the *TTN* 326 exon was affected the most. This may indicate that exon 326 represents an important functional domain. The *Clinvar* database contains 5762 records of different P/LP *TTN* variants (accessed in April 2026) [38], 1183 (20%) of which are located in the 326 exon which is the biggest exon of *TTN*. It contains 17,106 (15%) coding base pairs of the gene, while the average exon size is 314 base pairs. Counting only exons with PSI > 90%, it contains 21% base pairs of all these exons. Therefore, approximately one-fifth of the detected variants would be expected to occur in this exon. There is limited information on the evolutionary significance of such large exons. It may be speculated that larger exons could provide stability against alternative splicing.

Truncating *TTN* variants have been shown to be associated not only with DCM but also with atrial fibrillation and heart failure across different populations [39]. Some studies show that these variants lead to a more severe phenotype when additional environmental triggers are present [40]. Atrial fibrillation could be the first manifestation of DCM, showing a possible arrhythmogenic substrate [34]. We did not have data on whether atrial fibrillation was diagnosed before or after DCM diagnosis, but when analyzing if patients started seeking medical attention due to arrhythmia or other causes, we did not find differences in the *TTN*-positive group.

### Limitations

This study overviews our data regarding patients with DCM. We acknowledge that not all data are equally comparable. We did not find significant differences in heart ultrasound parameters between patients with positive and negative genetic findings, which could be due to the different specialists who performed the heart ultrasound. In this retrospective study, we did not have the opportunity to have all images evaluated by the same specialist. We lack comprehensive data regarding additional disorders or conditions that could have affected our comparison.

## 5. Conclusions

Genetic testing is important in DCM cases with a non-genetic cause as it allows us to establish diagnoses, especially in ambiguous cases. Despite not identifying clear clinical distinction between cases with and without causative genetic variants, in rare cases, finding a genetic cause allows better disorder control as with *BAG3* or *LMNA* variants. Furthermore, the identification of a genetic cause enables cascade testing of family members and facilitates earlier surveillance and disease management. Although our sample size is relatively small, the cohort may be broadly representative of a substantial proportion of the Lithuanian population, as LSMUL Kauno Klinikos is the country’s largest tertiary referral center. Comparable data are still lacking from centers in other countries.

## Figures and Tables

**Table 1:** Patients’ statistics.

Tested patients, *n*	169
Males	69.8% (*n* = 118)
Age at testing	51.55 (min 18; max 76.67; standard deviation 12.26)
Pathogenic variant identified	20.7% (*n* = 35)
Positive family history	21.3% (*n* = 36)
Reason for referral:	
arrhythmia	19.5% (*n* = 33)
shortness of breath	52.7% (*n* = 89)
prophylactic finding or nonspecific	27.8% (*n* = 47)

**Table 2:** Clinical and echocardiographic data.

	Negative	Positive	*p* Value
Males	69.4%, *n* = 93	71.4%, *n* = 25	0.816
Age at testing	52.05 ± 12.34	49.63 ± 11.93	0.19
Age at diagnosis (*n* = 164)	49.08 ± 14.17	47.32 ± 11.7	0.213
Affected family members	19.8%, *n* = 26	28.6%, *n* = 10	0.266
US LVEF (%)	30.68 ± 11.65	31.30 ± 13.10	0.837
US LVMMi (g/m^2^)	129.77 ± 34.58	123.13 ± 22.45	0.615
US LVEDDi (mm/m^2^)	30.69 ± 4.70	31.17 ± 3.36	0.318
MRT LVEF (%)	34.08 ± 11.40	32.01 ± 11.20	0.533
MRT LVEDVi (mL/m^2^)	140.57 ± 52.00	138.23 ± 30.53	0.49
MRT LVMMi (g/m^2^)	98.00 ± 30.31	95.50 ± 22.34	0.832
AF	40.3%, *n* = 54	40.0%, *n* = 14	0.974
VF/VT	14.9%, *n* = 20	14.3%, *n* = 5	0.924
Death	8.2%, *n* = 11	8.6%, *n* = 3	0.945
Implantable device	29.1%, *n* = 39	31.4%, *n* = 11	0.789

Patients were divided into groups based on positive (pathogenic or likely pathogenic causative genetic variant found) and negative (no causative variants found) genetic testing results. US LVEF—ultrasound left ventricle ejection fraction; US LVMMi—ultrasound left ventricle mass indexed; US LVEDDi—ultrasound left ventricle end-diastolic diameter indexed; MRT LVEF—magnetic resonance tomography left ventricle ejection fraction; MRT LVEDVi—magnetic resonance tomography left ventricle end-diastolic volume indexed; MRT LVMMi—magnetic resonance tomography left ventricle mass indexed; AF—atrial fibrillation; VF/VT—ventricle fibrillation/tachycardia; death—death within 5 years; implantable device—electrocardiostimulator or cardioverter defibrillator or cardiac resynchronization therapy.

**Table 3:** List of all pathogenic variants detected.

Gene	Nucleotide Change	Amino Acid Change	Affect	Exon/Intron	Novel	Initially Reported	Variant Class	Criteria Applied	Gender	Affected Family Members	Age at Diagnosis
*BAG3*	c.514C>T	p.Gln172*	Nonsense	3 exon	No	Yes	LP	PVS1s, PM2m	M	1	22
*TTN*	c.3034C>T	p.Arg1012*	Nonsense	18 exon	No	No	LP	PVS1s, PM2m, PS4s	M	0	61
*TTN*	c.9610C>T	p.Arg3204*	Nonsense	41 exon	No	No	LP	PVS1s, PM2m, PS4s	M	0	48
*TTN*	c.13696C>T	p.Gln4566*	Nonsense	48 exon	No	No	LP	PVS1s, PS4s	M	0	43
*TTN*	c.13696C>T	p.Gln4566*	Nonsense	48 exon	No	No	LP	PVS1s, PS4s	M	0	61
*TTN*	c.13696C>T	p.Gln4566*	Nonsense	48 exon	No	No	LP	PVS1s, PS4s	M	0	58
*TTN*	c.13696C>T	p.Gln4566*	Nonsense	48 exon	No	Yes	LP	PVS1s, PS4s	M	1	48
*TTN*	c.13696C>T	p.Gln4566*	Nonsense	48 exon	No	No	LP	PVS1s, PS4s	M	0	unknown
*LMNA*	c.961C>T	p.Arg321*	Nonsense	6 exon	No	Yes	P	PVS1vs, PM2m, PS4s	M	1	40
*TTN*	c.43158_43162del	p.Glu14387Phefs*6	Frameshift	234 exon	Yes	No	LP	PVS1s, PM2m	M	0	40
*TTN*	c.45322C>T	p.Arg15108*	Nonsense	245 exon	Yes	Yes	LP	PVS1s, PM2m, PS4s	M	1	64
*TTN*	c.46047_46048del	p.Tyr15349*	Nonsense	248 exon	Yes	Yes	LP	PVS1s, PM2m	M	1	33
*BAG3*	c.1385T>C	p.Leu462Pro	Missense	4 exon	No	Yes	VUS	PP3p, PM2m, PS4m	M	0	44
*FLNC*	c.1389delC	p.Phe464Serfs*28	Frameshift	8 exon	Yes	Yes	LP	PVS1s, PM2m	F	0	44
*TTN*	c.47203A>T	p.Arg15735*	Nonsense	252 exon	Yes	Yes	LP	PVS1s, PM2m	M	1	44
*TTN*	c.57165del	p.Gly19056Glufs*28	Frameshift	293 exon	Yes	Yes	LP	PVS1s, PM2m	F	1	46
*TTN*	c.58859del	p.Thr19620Ilefs*26	Frameshift	299 exon	Yes	Yes	LP	PVS1s, PM2m	F	0	51
*TTN*	c.59201_59202del	p.Pro19734fs	Frameshift	300 exon	No	Yes	LP	PVS1s, PM2m, PS4s	M	0	58
*TTN*	c.64554_64556del	p.Ser21520Glnfs*12	Frameshift	309 exon	Yes	Yes	LP	PVS1s, PM2m	F	1	39
*TTN*	c.67024A>T	p.Lys22342*	Nonsense	317 exon	Yes	Yes	LP	PVS1s, PM2m	M	0	70
*TTN*	c.85090C>T	p.Arg28364*	Nonsense	326 exon	No	Yes	P	PVS1s, PM2m, PS4s	F	0	31
*TTN*	c.69923_69926dup	p.Lys23309Asnfs*2	Frameshift	326 exon	No	Yes	LP	PVS1s, PM2m	F	0	34
*TTN*	c.69923_69926dup	p.Lys23309Asnfs*2	Frameshift	326 exon	No	Yes	LP	PVS1s, PM2m	F	0	42
*DSP*	c.2130+1G>A	Not applicable	Splice site	15 intron	No	Yes	LP	PVS1m, PM2m, PS1m	F	0	56
*TTN*	c.81679G>T	p.Glu27227*	Nonsense	326 exon	Yes	Yes	LP	PVS1s, PM2m	F	1	49
*TTN*	c.69923_69926dup	p.Lys23309Asnfs*2	Frameshift	326 exon	No	Yes	LP	PVS1s, PM2m	M	0	56
*TTN*	c.69923_69926dup	p.Lys23309Asnfs*2	Frameshift	326 exon	No	Yes	LP	PVS1s, PM2m	M	0	61
*TTN*	c.84504dup	p.Ser28169Ilefs*12	Frameshift	326 exon	No	Yes	LP	PVS1s, PM2m	M	0	58
*TTN*	c.79568del	p.Val26523Glufs*2	Frameshift	326 exon	Yes	Yes	LP	PVS1s, PM2m	M	0	29
*TTN*	c.85090C>T	p.Arg28364*	Nonsense	326 exon	No	Yes	LP	PVS1s, PM2m	M	0	29
*TTN*	c.82325dup	p.Arg27443Profs*7	Frameshift	326 exon	Yes	Yes	LP	PVS1s, PM2m	M	0	51
*TTN*	c.78246C>A	p.Cys26082*	Nonsense	326 exon	Yes	Yes	LP	PVS1s, PM2m	M	0	65
*TTN*	c.95341C>T	p.Arg31781*	Nonsense	343 exon	No	Yes	LP	PVS1s, PM2m, PS4s, PM6m	M	0	49
*TTN*	c.99672del	p.Val33225Tyrfs*4	Frameshift	355 exon	Yes	Yes	LP	PVS1s, PM2m	F	0	37
*TTN*	c.51436+1G>C	Not applicable	Splice site	271 intron	Yes	No	LP	PVS1s, PM2m	M	1	48

Patients’ data by genetic variant identified. P—pathogenic; LP—likely pathogenic; VUS—variant of unknown significance. “Criteria Applied”—rules used by ACMG 2015 [21] and Clingen [22]. M—male; F—female. The column “Novel” shows if variants were previously published in the literature or databases. “Initially Reported” shows if variants were previously reported for the patient or they were classified as P/LP only in this study.

## Data Availability

The original contributions presented in this study are included in the article/Supplementary Materials. Further inquiries can be directed to the corresponding author: marius.sukys@lsmu.lt.

## Supplementary Materials

The following supporting information can be downloaded at: https://www.mdpi.com/article/10.3390/diagnostics16132115/s1, List S1: Gene panel used for the samples until May 2020; List S2: Gene panel used after May 2020.

## Abbreviations

The following abbreviations are used in this manuscript:

ACMG	American College of Medical Genetics
AF	Atrial Fibrillation
AMP	Association for Molecular Pathology
DCM	Dilated Cardiomyopathy
ECG	Electrocardiography
LV	Left Ventricle
LVEDDi	Left Ventricular End-Diastolic Diameter Indexed
LVEDVi	Left Ventricular End-Diastolic Volume Indexed
LVEF	Left Ventricular Ejection Fraction
LVMMi	Left Ventricular Mass Indexed
MRI	Magnetic Resonance Imaging
P/LP	Pathogenic/Likely Pathogenic
PSI	Percentage Spliced-In
TTE	Transthoracic Echocardiography
VF	Ventricular Fibrillation
VT	Ventricular Tachycardia
VUS	Variant of Uncertain Significance
